# Reply to Mazela et al. Comment on “Jankiewicz et al. The Effect of Goat-Milk-Based Infant Formulas on Growth and Safety Parameters: A Systematic Review and Meta-Analysis. *Nutrients* 2023, *15*, 2110”

**DOI:** 10.3390/nu15214559

**Published:** 2023-10-27

**Authors:** Mateusz Jankiewicz, Lucie van der Zee, Hania Szajewska

**Affiliations:** 1 Department of Paediatrics, The Medical University of Warsaw, 02-091 Warsaw, Poland; 2 Ausnutria B.V., 8025 BM Zwolle, The Netherlands

Thank you for carefully reading and commenting [1] on our review entitled “The effect of goat-milk-based infant formulas on growth and safety parameters: a systematic review and meta-analysis” [2].

As you indicated in your comment, the formula used by Grant et al. [3] and Zhou et al. [4] contains both proteins and fat from whole milk. However, this information is not specified in Grant et al. [3] and was therefore not mentioned in our review. We acknowledge that the formula evaluated by Xu et al. [5] and He et al. [6] is based on skim milk powder and whey protein powder enriched with a vegetable oil blend as a source of fat. However, according to our findings, these differences had no impact on the outcomes evaluated in our review. Neither anthropometric parameters nor stool frequency differences were statistically significant between the goat milk-based formulas that were evaluated and cow milk-based formulas [2]. Furthermore, when compared to human milk, both formulas tested by Zhou et al. [4] and He et al. [6] showed lower stool frequency in infants, with no significant differences between them.

We agree that the introduction should contain information about the lower αS1-casein level as linked with the lower aggregation of proteins instead of a higher αS2-casein level. The randomization process in Zhou et al.’s study [4] was assessed as “some concerns” according to the guidelines from the Cochrane Handbook for Systematic Reviews of Interventions [7] and using Cochrane Collaboration’s tool [8] due to significant differences between the intervention groups in baseline. Table 1 in the original article [2] does not contain data from the study published by Tannock et al. [9], because that study did not meet the inclusion criteria for this review. We agree that Table 1 has an error about the intervention period in Zhou et al.’s work [4] (12 months versus 4–12 months). Furthermore, Grant et al.’s work [3] was marked as “no published study protocol available” due to the lack of information in the article about published protocol. It only contains a description of the protocol in the final article. Finally, we acknowledge that the company authors should be mentioned in the Conflicts of Interest section. However, the article does include the information that authors Linde van Lee and Lucie van der Zee are employees of Ausnutria B.V.

Apart from that, the conclusions from the metanalysis should be correct. Our systematic review showed that randomized controlled trials support goat milk-based formulas as a safe and well-tolerated alternative to cow milk-based formulas, irrespective of their compositional differences.
